# Identification of cross-talk pathways and PANoptosis-related genes in periodontitis and Alzheimer’s disease by bioinformatics analysis and machine learning

**DOI:** 10.3389/fnagi.2024.1430290

**Published:** 2024-08-27

**Authors:** Xiantao Chen, Yifei Dai, Yushen Li, Jiajun Xin, Jiatong Zou, Rui Wang, Hao Zhang, Zhihui Liu

**Affiliations:** ^1^Hospital of Stomatology, Jilin University, Changchun, China; ^2^Jilin Provincial Key Laboratory of Tooth Development and Bone Remodeling, Changchun, China

**Keywords:** periodontitis, Alzheimer’s disease, PANoptosis, common genes, immune infiltration

## Abstract

**Background and objectives:**

Periodontitis (PD), a chronic inflammatory disease, is a serious threat to oral health and is one of the risk factors for Alzheimer’s disease (AD). A growing body of evidence suggests that the two diseases are closely related. However, current studies have not provided a comprehensive understanding of the common genes and common mechanisms between PD and AD. This study aimed to screen the crosstalk genes of PD and AD and the potential relationship between cross-talk and PANoptosis-related genes. The relationship between core genes and immune cells will be analyzed to provide new targets for clinical treatment.

**Materials and methods:**

The PD and AD datasets were downloaded from the GEO database and differential expression analysis was performed to obtain DEGs. Overlapping DEGs had cross-talk genes linking PD and OP, and PANoptosis-related genes were obtained from a literature review. Pearson coefficients were used to compute cross-talk and PANoptosis-related gene correlations in the PD and AD datasets. Cross-talk genes were obtained from the intersection of PD and AD-related genes, protein-protein interaction(PPI) networks were constructed and cross-talk genes were identified using the STRING database. The intersection of cross-talk and PANoptosis-related genes was defined as cross-talk-PANoptosis genes. Core genes were screened using ROC analysis and XGBoost. PPI subnetwork, gene-biological process, and gene-pathway networks were constructed based on the core genes. In addition, immune infiltration on the PD and AD datasets was analyzed using the CIBERSORT algorithm.

**Results:**

366 cross-talk genes were overlapping between PD DEGs and AD DEGs. The intersection of cross-talk genes with 109 PANoptosis-related genes was defined as cross-talk-PANoptosis genes. ROC and XGBoost showed that MLKL, DCN, IL1B, and IL18 were more accurate than the other cross-talk-PANoptosis genes in predicting the disease, as well as better in overall characterization. GO and KEGG analyses showed that the four core genes were involved in immunity and inflammation in the organism. Immune infiltration analysis showed that B cells naive, Plasma cells, and T cells gamma delta were significantly differentially expressed in patients with PD and AD compared with the normal group. Finally, 10 drugs associated with core genes were retrieved from the DGIDB database.

**Conclusion:**

This study reveals the joint mechanism between PD and AD associated with PANoptosis. Analyzing the four core genes and immune cells may provide new therapeutic directions for the pathogenesis of PD combined with AD.

## 1 Introduction

Periodontitis (PD) is a chronic inflammatory disease with a high prevalence, as high as 50% of the population over 30 years of age in the United States, making it the sixth most common disease in humans (Eke et al., 2018). It is caused by the interaction between oral bacteria and the host immune response (Konig et al., 2016). Without timely intervention, periodontitis can lead to tooth loss and serious oral health consequences (Kaur et al., 2018).

In recent years, with advances in periodontology, more and more scholars are exploring the potential neurological effects of PD. AD is a progressive neurodegenerative disease characterized by cognitive decline, memory loss, and behavioral and personality changes (Chen and Johansson, 2024). It is the most common form of dementia, affecting millions of people worldwide (Yuan et al., 2024). At the molecular level, AD is characterized by the development of amyloid beta (Aβ) plaques, neuroprogenitor fiber tangles, and neuroinflammation (Donohue et al., 2017). Cross-sectional analysis of a population-based study found that an increase in peripheral inflammatory markers, such as CRP, IL-1β, IL-6, and TNF-α, in AD patients is associated with an increase in the incidence of dementia in elderly patients (Gorelick, 2010). Results from epidemiological and translational studies have suggested that inflammation outside the CNS (i.e., systemic inflammation) may promote neurodegenerative and Alzheimer ’s-specific lesions within the brain (Walker et al., 2019), individuals with PD have a higher risk of developing AD than those without PD (Chen et al., 2017; Nadim et al., 2020; Dioguardi et al., 2020). In addition, many animal model experiments have shown that periodontitis may cause or directly exacerbate pathology, neuroinflammation, and cognitive function in patients with AD (Ishida et al., 2017; Ilievski et al., 2018; Kantarci et al., 2020). A number of studies have shown that patients with AD have poorer dental health than aged controls and that the more severe the dementia the worse the dental health (Harding and Singhrao, 2021). Although there is evidence to support a link between PD and AD, further research is needed to elucidate the exact mechanisms and establish a causal relationship.

It is noteworthy that PD, as a localized oral inflammation, is closely related to systemic inflammation (Hajishengallis, 2014). Recent studies have shown that bacteria or locally activated immune cells in the periodontal tissues of patients with PD can enter extraoral tissues. They cause systemic inflammation and contribute to a variety of chronic diseases such as cardiovascular diseases, neurodegenerative diseases, autoimmune diseases, and cancer (Gorelick, 2010; Hajishengallis, 2014; Genco and Sanz, 2020; Potempa et al., 2017). The effects of PD and systemic states may also be reciprocal. In this context, we may venture to hypothesize that inflammation may be an important mechanism for the link between the two diseases.

PANoptosis is a unique new form of programmed cell death (PCD), which, as a form of inflammatory cell death, involves not only the molecular components of pyroptosis and necroptosis but also apoptosis (Henkel and O’Neill, 2023; Qi et al., 2023). The important role of PANoptosis in PD has been confirmed by in vivo and in vitro experiments (Zhang A. et al., 2023; Liu et al., 2022). The link between AD and PANoptosis has not yet been experimentally demonstrated. Nevertheless, some scholars have recognized that PANoptosis may play a key role in the pathogenesis of AD, and the link between them deserves further exploration (Rajesh and Kanneganti, 2022). Therefore, investigating the relationship between PD and AD combined with PANoptosis could help to understand the pathophysiologic mechanisms behind its development and guide coordinated interdisciplinary management in the clinical setting.

We used bioinformatics to overcome the difficulties of joint clinical studies of PD and AD. By searching for cross-talk genes between PD and AD and linking them to cell death-related genes using correlation analysis and PPI networks, we hypothesized the key genes of the relationship between PD and AD and their associated signaling pathways, investigated the mechanisms of interactions between the two disorders, and proposed additional hypotheses for clinical research questions.

**FIGURE 1:**
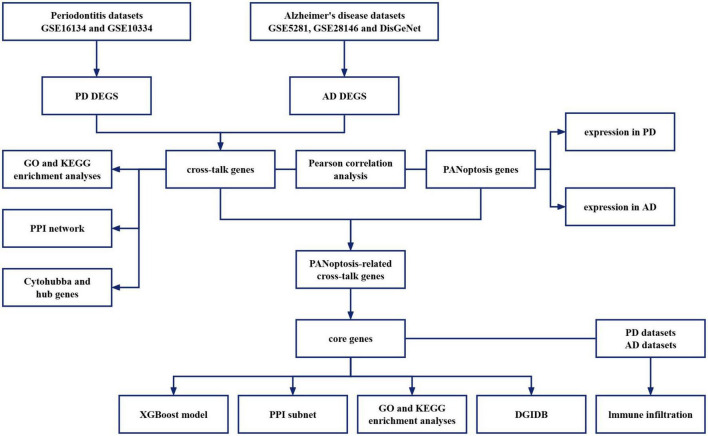
Flow-chart of datasets analysis in this paper.

This study reveals the potential mechanisms of PD combined with AD by exploring cross-talk and PANoptosis-related genes between PD and AD. Diagnostic markers were identified from common genes to investigate their association with immune infiltration and their potential as diagnostic biomarkers and therapeutic targets. The flowchart of the analysis is shown in Figure 1.

## 2 Materials and methods

### 2.1 Data downloading and preprocessing

Gene Expression Omnibus (GEO)^1^ is a public database of gene expression data, for the researchers to download and use.

PD datasets were searched using the keyword: “periodontitis”. Screening criteria: Datasets were derived from Homo sapiens, and the experimental type was microarray. Based on these criteria, we obtained two periodontitis-related datasets (GSE16134 and GSE10334). AD datasets were searched using the keyword: “Alzheimer’s disease”. The screening criteria were the same as PD. We obtained two Alzheimer’s disease-related datasets (GSE5281 and GSE28146). The data obtained from GEO is shown in Table 1.

**TABLE 1:** The information of datasets.

Datasets	Organism	Sample	Platform	Case	Control
GSE16134	Homo sapiens	Gingival tissue	GPL570	241	69
GSE10334	Homo sapiens	Gingival tissue	GPL570	183	64
GSE5281	Homo sapiens	Brain tissue	GPL570	87	74
GSE28146	Homo sapiens	Brain tissue	GPL570	22	8

Based on the information from the datasets, intersecting genes were obtained between each disease dataset. PD dataset and AD dataset were merged respectively, and the combat method of the sva package (version 3.44.0) in R software (version 4.2.2) was used to correct the merged dataset to remove the batch effect.

### 2.2 Identification of differentially expressed genes

After batch correction, the limma package (version 3.52.4) was used to analyze the differential expression between case samples and healthy samples in the merged datasets. In PD, DEGs were screened with the threshold of *P*-Value < 0.05 and | log fold change (FC) | > 0.5. In AD, DEGs were also screened with the threshold of *P*-Value < 0.05 and | log fold change (FC) | > 0.5. The expression of DEGs was demonstrated by the ggpubr package (version 0.6.0), ggthemes package (version 5.1.0), and pheatmap package (version 1.0.12) with volcano plots and heatmaps.

### 2.3 Functional and pathway enrichment analysis of DEGs

Gene Ontology (GO) analysis, the most used bioinformatics tool for gene annotation, covers three types of gene functions: BP, molecular function (MF), and cellular component (CC). The Kyoto Encyclopedia of Genes and Genomes provided details on genomes, biological pathways, diseases, and chemicals. We uploaded the DEGs of the PD and AD to the Metascape database (Zhou et al., 2019). The *P*-Value < 0.01, Min Overlap = 3, and Min Enrichment = 1.5 were selected as the threshold for GO and KEGG pathway enrichment analyses.

### 2.4 Identification and enrichment analysis of cross-talk genes

DisGeNET^2^ is a database of genes related to diseases. We used “Alzheimer’s disease” as the keyword in the DisGeNet database for text mining to obtain AD-related genes. To make the results of the subsequent analysis more comprehensive and accurate, AD-related genes were merged into AD DEGs.

The DEGs of PD and AD were identified by R software, and they were imported into the R software to obtain the intersection of PD DEGs and OP DEGs. These commonly dysregulated genes in PD and OP may be keys to the link between the two, which we call cross-talk genes. Then the cross-talk genes were uploaded to the Metascape database for enrichment analysis. The threshold was *P*-value < 0.01, Min Overlap = 3 and Min Enrichment = 1.5.

### 2.5 Protein-protein interaction network construction and hub gene identification

String^3^ is a database that can be used to retrieve known and predicted protein-protein interactions (Szklarczyk et al., 2023). To obtain the PPI network, the cross-talk genes were imported into the String database. The minimum required interaction score was set to 0.4 to mine the interactions between protein-coding genes. Then, the PPI network downloaded from the String database was analyzed by Cytoscape software (version 3.8.0) (Shannon et al., 2003). CytoHubba is a plugin in Cytoscape. Using the methods (Degree, EPC, MCC, and MNC) of the cytohubba to identify hub genes in the PPI network. The hub genes were uploaded to the Metascape database for enrichment analysis. The *P*-value < 0.01, Min Overlap = 3, and Min Enrichment = 1.5 were selected as the threshold for GO and KEGG pathway enrichment analyses.

### 2.6 Correlation analysis between cross-talk genes and PANoptosis-related genes

Through a literature search, 109 genes involved in the PANoptosis process were collected from previous studies, as detailed in Table 2 (Wei et al., 2023; Chen G. et al., 2022; Zhuang et al., 2023; Abulaiti et al., 2023; Huang et al., 2022; Wang et al., 2022). To study the potential role of PANoptosis between PD and AD, we obtained the expression profiles of cross-talk genes and PANoptosis-related genes in PD and AD datasets. Pearson correlation analysis was performed using the Hmisc package (version 5.0-1). We calculated Pearson correlation coefficient (r) values and screened genes with moderate and strong correlation (*P*-value < 0.05, | r| > 0.5). Input the calculated results into eGPS software (version 1.23) to generate a heatmap of the correlation between cross-talk genes and PANoptosis-related genes.

**TABLE 2:** PANoptosis-related genes from literature.

	Genes
PANoptosis	ADAR, MEFV, AIM2, NLRP1, NLRC4, NLRP9, NLRP3, ZBP1, TNFRSF1A, PYCARD, FADD, CASP10, CASP1, CASP2, CASP12, CASP4, CASP3, CASP6, CASP5, CASP7, DFNA5, CASP8, MLKL, GSDMD, RIPK3, RIPK1, NAIP, TNF, NLRP6, GSDMA, GSDMC, GSDMB, APAF1, BAK, BAX, DIABLO, DCN, CASP9, FAS, TLR3, FASLG, BAK1, CHMP2A, CHMP2B, CHMP3, CHMP4A, CHMP4B, CHMP4C, CHMP6, CHMP7, CYCS, ELANE, GSDME, GZMB, HMGB1, IL18, IL1A, IL1B, IRF1, IRF2, TP63, AIFM1, AKT3, APPL1, BMF, BNIP3L, BOK, CD14, CHUK, CRADD, DFFA, DFFB, E2F1, HMGB2, IGF1, LY96, PPP3R1, TFDP1, TICAM1, TNFSF10, TP73, TRAF2, UACA, UNC5B, YWHAE, YWHAG, MAP3K7, TNFAIP3, RNF31, RBCK1, PSTPIP2, TAB3, PARP1, TRADD, TAB2, NR2C2, AXL, CFLAR, GPX4, GZMA, IL6, NLRP7, NOD1, PLCG1, PRKACA, TLR4, TNFRSF10A, TNFRSF10B, TP53

### 2.7 Discovery of cross-talk- PANoptosis genes

The cross-talk genes and PANoptosis-related genes were imported into the R software. The intersection of cross-talk genes and PANoptosis-related genes was defined as PANoptosis-related cross-talk genes, and these genes deserve to be explored in the shared mechanism of PD and AD. These genes would be used for subsequent analysis.

### 2.8 Receiver operating characteristic curve analysis

To further explore the importance of PANoptosis-related cross-talk genes as potential biomarkers, the pROC package (version 1.18.0) was used for receiver operating characteristic (ROC) curve analysis of PANoptosis-related cross-talk genes in two diseases, and the calculation of area under the curve (AUC) was performed to quantify its value. In PD, genes with AUC > 0.75 were considered to be meaningful. In AD, genes with AUC > 0.55 were considered to be meaningful.

### 2.9 Identification of core genes

The intersection of the above genes was defined as the core genes. To further study the role of core genes as a whole in these two diseases, we defined them as a whole as the key signature. We used the caret package (version 6. 0–94) to divide the PD dataset and AD dataset into training and testing sets at the 7:3 ratio. The xgboost package (version 1.7.5.1), a machine-learning method, was used to construct the classification model, and the importance score of each feature was viewed by “xgb.ggplot.importance” function of xgboost package (version 6. 0–94).

### 2.10 Analysis of biological processes and pathways of core genes

We used Cytoscape software (version 3.8.0) to extract the PPI subnet associated with core genes from the previous PPI network. Then, we imported core genes into the GO database (Carbon et al., 2009) and KEGG database (Kanehisa and Goto, 2000). We collected biological processes and pathways that contain at least two core genes to obtain the potential association between core genes and pathways, and between pathways and pathways.

### 2.11 Immune infiltration

We used the CIBERSORT (Newman et al., 2015) algorithm in R software to obtain immune infiltration matrixes from PD and AD gene expression datasets. Wilcoxon test was used to compare the differences between the two groups. Then, we used the corrplot package (version 0.92) to plot correlation heatmaps to visualize the correlation between the 22 infiltrating immune cells and the correlation between core genes and immune cells.

### 2.12 Drug-gene interaction analysis

DGIDB^4^ is a database of drug-gene interactions. To screen potential drugs targeting core genes, We uploaded core genes to the DGIDB database. Drugs with an interaction score > 2.5 were defined as potentially effective.

## 3 Results

### 3.1 Data preprocessing

**FIGURE 2:**
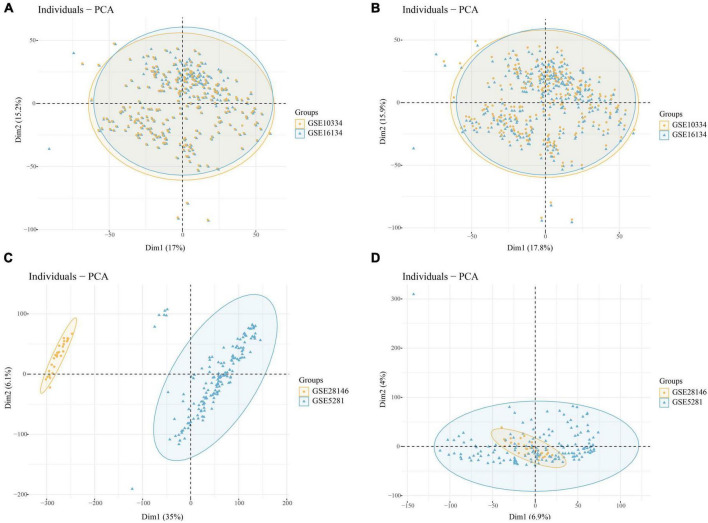
**(A)** PCA analysis results of PD datasets before batch correction; **(B)** PCA analysis results of PD datasets after batch correction; **(C)** PCA analysis results of AD datasets before batch correction; **(D)** PCA analysis results of AD datasets after batch correction.

Through batch correction, PD datasets (GSE16134 and GSE10334) and AD datasets (GSE5281 and GSE28146) were obtained. The PD datasets contained 424 case samples and 133 control samples, while the AD datasets contained 109 case samples and 82 control samples. Figure 2A shows that the PD datasets (GSE16134 and GSE10334) differ. Figure 2C shows that the AD dataset (GSE5281 and GSE28146) differed significantly. Figures 2B, D show that the batch effect from the combined dataset was significantly eliminated. Through PCA analysis, it was found that the differences between datasets were significantly reduced (Figure 2).

### 3.2 Identification and enrichment analysis of DEGs

**FIGURE 3:**
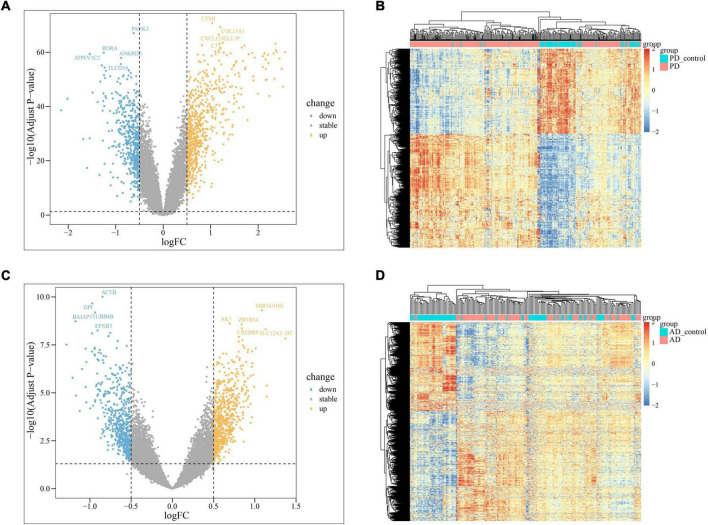
**(A)** The volcano plots of PD DEGs; **(B)** the heatmap of PD up-regulated and down-regulated genes; PD present the samples of the monocytes from osteoporosis patient and PD_control present the samples of the monocytes from non-osteoporotic patients; **(C)** the volcano plots of AD DEGs; **(D)** the heatmap of AD up-regulated and down-regulated genes; AD present the samples of the monocytes from osteoporosis patient and AD_control present the samples of the monocytes from non-osteoporotic patients.

**FIGURE 4:**
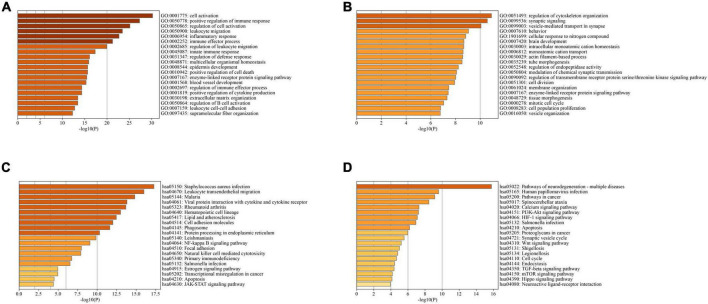
**(A)** TOP20 GO BP terms of PD DEGs; **(B)** TOP20 GO BP terms of AD DEGs; **(C)** TOP20 KEGG pathways of PD DEGs; **(D)** TOP20 KEGG pathways of AD DEGs.

In PD and AD datasets, differential gene analysis was performed, resulting in 697 up-regulated genes and 520 down-regulated genes in PD, and 700 up-regulated genes and 563 down-regulated genes in AD. The expression of DEGs in PD and AD datasets was depicted using heatmaps (Figures 3B, D). Volcano plots were utilized to show the distribution of DEGs in PD datasets (Figure 3A) and AD datasets (Figure 3C). The top 5 up-regulated and down-regulated DEGs with the most significant P-value were labeled. GO and KEGG results indicated that DEGs in both PD and AD were involved in “Apoptosis” and “Salmonella infection” (Figure 4). Studies have shown that “Salmonella infection” is associated with pyroptosis (Akhade et al., 2020). PANoptosis is a specific programmed cell death mechanism that includes apoptosis, pyroptosis, and necroptosis, with extensive interactions among them (Zheng et al., 2020). Therefore, the results confirm that PANoptosis is involved in the common pathogenesis of PD and AD.

### 3.3 Identification and enrichment analysis of cross-talk genes

**FIGURE 5:**
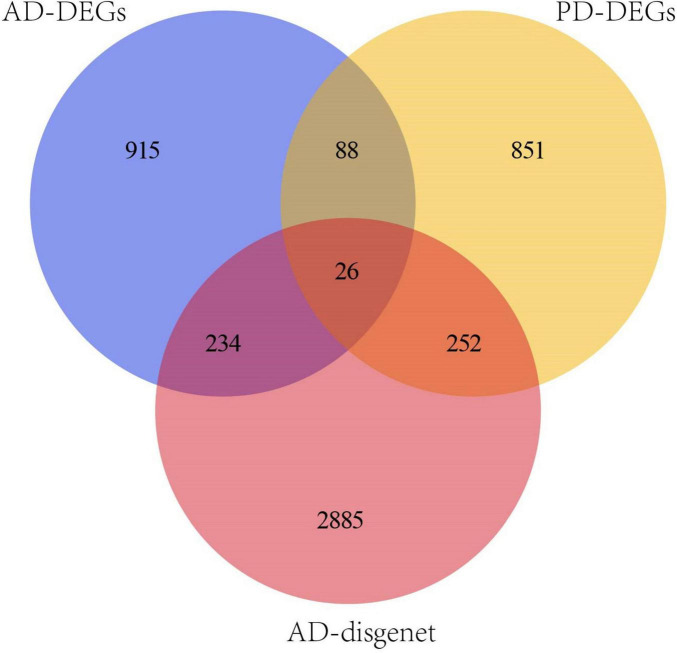
Venn diagram of the intersection of PD DEGs,OP DEGs and AD-disgenet.

**FIGURE 6:**
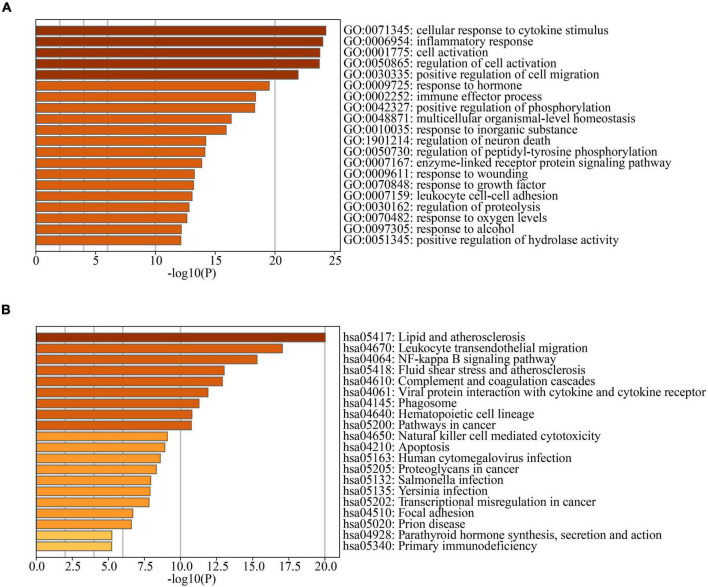
**(A)** TOP20 GO BP terms of cross-talk genes; **(B)** TOP20 KEGG pathways of cross-talk genes.

3397 AD-related genes were obtained from the DisGeNet database and merged into AD DEGs. The intersection with PD DEGs resulted in 366 cross-talk genes (Figure 5). The Metascape database was used to identify the BPs and KEGG pathways enriched by 366 cross-talk genes. Enrichment analysis revealed that these cross-talk genes were primarily enriched in several biological processes, including “cellular response to cytokine stimulus,” “inflammatory response”, “cell activation”, “regulation of cell activation”, and “immune effector process” (Figure 6A). Additionally, these cross-talk genes were also enriched in pathways such as “Lipid and atherosclerosis”, “Leukocyte transendothelial migration”, “Viral protein interaction with cytokine and cytokine receptor” and “Apoptosis” (Figure 6B).

### 3.4 PPI network analysis of cross-talk genes and hub gene identification

**FIGURE 7:**
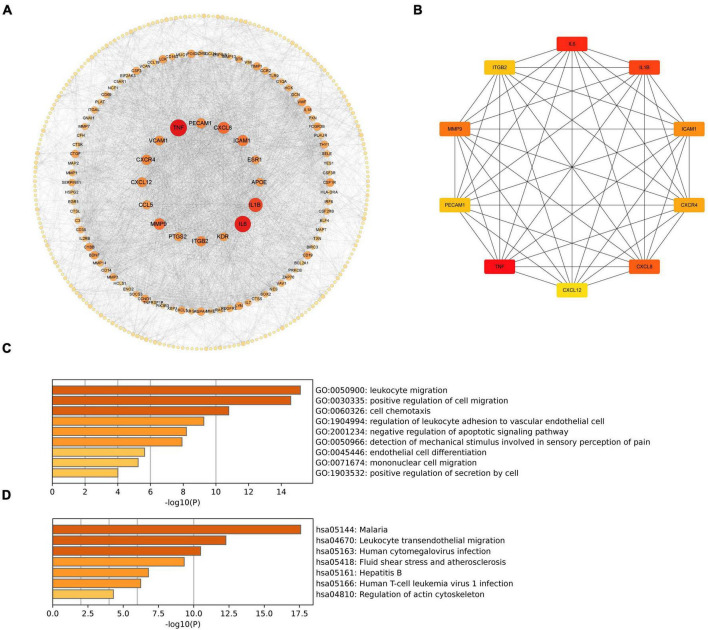
**(A)** PPI network of cross-talk genes; **(B)** CytoHubba analysis of PPI network with Cytoscape; **(C)** TOP9 GO BP terms of hub genes; **(D)** TOP7 KEGG pathways of hub genes.

To investigate the associations of cross-talk genes, we constructed a Protein-Protein Interaction (PPI) network using Cytoscape and the STRING database (Figure 7A) and identified ten hub genes (Figure 7B). Figure 7C illustrates that the biological functions of hub genes are enriched in processes such as “leukocyte migration,” “cell chemotaxis”, “regulation of leukocyte adhesion to vascular endothelial cell”, “negative regulation of apoptotic signaling pathway” and “mononuclear cell migration.” Furthermore, Figure 7D shows that the pathways associated with hub genes are primarily enriched in “Malaria”, “Leukocyte transendothelial migration”, “Human cytomegalovirus infection”, “Fluid shear stress and atherosclerosis”, “Hepatitis B”, “Human T-cell leukemia virus 1 infection” and “Regulation of actin cytoskeleton”.

These results suggest that hub genes may influence both PD and AD by regulating processes such as cell migration, immune responses, and programmed cell death.

### 3.5 Correlation analysis between cross-talk genes and PANoptosis-related genes

**FIGURE 8:**
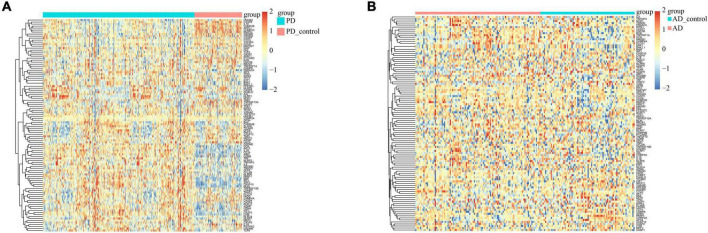
**(A)** Heatmap of PANoptosis-related genes expression in PD samples; **(B)** Heatmap of PANoptosis-related genes expression in AD samples.

**FIGURE 9:**
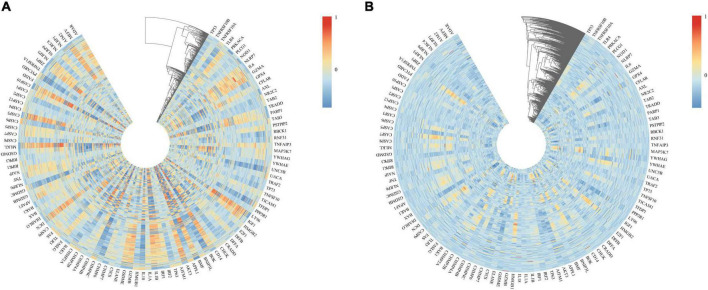
**(A)** Heatmap of the correlation between cross-talk and PANoptosis-related genes in PD samples; **(B)** Heatmap of the correlation between cross-talk and PANoptosis-related genes in AD samples.

We used heatmaps to depict the expression of 109 PANoptosis-related genes in both diseases. Red rectangles represent high expression, and blue rectangles represent low expression (Figure 8). Figures 8A, B show that most of the PANoptosis-related genes were highly expressed in PD and AD, suggesting that PANoptosis is involved in the development of both diseases. Pearson correlation coefficients were calculated to analyze the correlation between 109 PANoptosis-related genes and 366 cross-talk genes in the two diseases separately. The colors of the squares represent the strength of the correlation; red represents a positive correlation, and blue represents a negative correlation. The darker the color, the stronger the correlation (Figure 9). In PD, we identified a total of 2165 gene pairs with significant correlations, while in AD, we identified 89 gene pairs (P-value < 0.05, | r| > 0.5). These results confirm the involvement of PANoptosis in the cross-talk pathways of both PD and AD.

### 3.6 Identification of cross-talk-PANoptosis genes

The intersection of cross-talk genes and PANoptosis-related genes yielded a total of 9 cross-talk-PANoptosis genes, which include AIM2, CD14, DCN, GZMB, IL1B, IL18, IL6, MLKL and TNF.

### 3.7 ROC curve analysis

**FIGURE 10:**
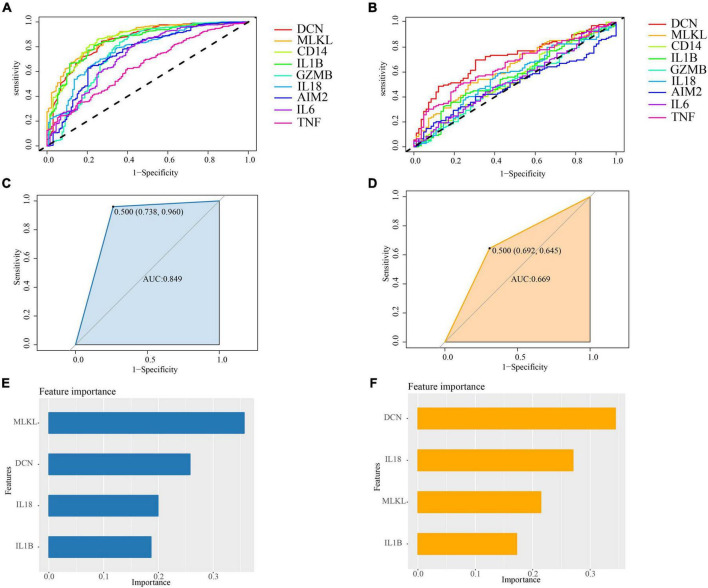
**(A)** ROC curve analysis of cross-talk-PANoptosis genes in PD samples; **(B)** ROC curve analysis of cross-talk-PANoptosis genes in AD samples; **(C)** the ROC of XGBoost in PD samples. The AUC is 0.849; **(D)** the ROC of XGBoost in AD samples. The AUC is 0.669; **(E)** the importance of features in PD samples; **(F)** the importance of features in OP samples.

As shown in Figure 10, we have identified 6 genes in PD, namely MLKL (AUC = 0.8715), CD14 (AUC = 0.8579), DCN (AUC = 0.8495), IL1B (AUC = 0.8459), IL18 (AUC = 0.7755), and GZMB (AUC = 0.7581). In AD, we have selected 5 genes, which are DCN (AUC = 0.7075), TNF (AUC = 0.6516), MLKL (AUC = 0.6304), IL1B (AUC = 0.5638), and IL18 (AUC = 0.5626) (Figures 10A, B). Therefore, we have chosen DCN, MLKL, IL1B, and IL18 as the core genes.

To further investigate the overall impact of these core genes in both diseases, we built a model using XGBoost. The results indicate that the classification efficiency in PD is 0.849, while in AD, it is 0.669 (Figures 10C, D). Hence, we conclude that the core genes play a significant role in the onset and progression of both PD and AD, and there exist interactions among these genes.

The XGBoost model can calculate importance scores for each gene and rank them. By examining the importance score of each feature, we can understand the impact of each feature on the model. In our model, DCN is a relatively important feature in both diseases (Figures 10E, F).

### 3.8 Analysis of biological processes and pathways of core genes

**FIGURE 11:**
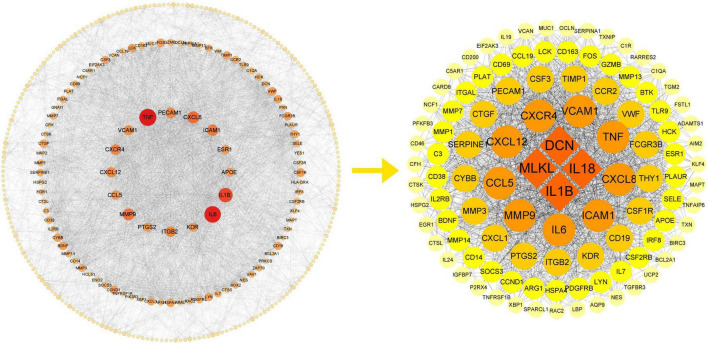
PPI subnetwork of core genes.

To gain a clearer understanding of the interactions between core genes, we extracted a subnetwork from the previous PPI network composed of core genes and genes directly related to the core genes (Figure 11).

Uploading the core genes to the GO database, we obtained 241 biological processes that involve at least two core genes, (Table 3). Among them, processes such as “positive regulation of T-helper 1 cell cytokine production”, “positive regulation of granulocyte-macrophage colony-stimulating factor production”, “positive regulation of neuroinflammatory response” and “lipopolysaccharide-mediated signaling pathway” were considered important.

**TABLE 3:** GO analysis of core genes.

	GO biological process complete	SUM
1	Biological regulation (GO:0065007)	4
2	Cellular process (GO:0009987)	4
3	Regulation of cellular process (GO:0050794)	4
4	Regulation of biological process (GO:0050789)	4
5	Biological_process (GO:0008150)	4
6	Regulation of primary metabolic process (GO:0080090)	3
7	Defense response (GO:0006952)	3
8	Response to stress (GO:0006950)	3
9	Positive regulation of RNA biosynthetic process (GO:1902680)	3
10	Nitrogen compound metabolic process (GO:0006807)	3
11	Regulation of metabolic process (GO:0019222)	3
12	Regulation of nucleobase-containing compound metabolic process (GO:0019219)	3
13	Regulation of macromolecule metabolic process (GO:0060255)	3
14	Positive regulation of intracellular signal transduction (GO:1902533)	3
15	Regulation of intracellular signal transduction (GO:1902531)	3
16	Organic substance metabolic process (GO:0071704)	3
17	Regulation of RNA biosynthetic process (GO:2001141)	3
18	Cell surface receptor signaling pathway (GO:0007166)	3
19	Signal transduction (GO:0007165)	3
20	Cell communication (GO:0007154)	3
21	Regulation of transcription by RNA polymerase II (GO:0006357)	3
22	Regulation of DNA-templated transcription (GO:0006355)	3
23	Cellular response to stimulus (GO:0051716)	3
24	Response to other organism (GO:0051707)	3
25	Developmental process (GO:0032502)	3
26	Organonitrogen compound metabolic process (GO:1901564)	3
27	Anatomical structure development (GO:0048856)	3
28	Biological process involved in interspecies interaction between organisms (GO:0044419)	3
29	Regulation of multicellular organismal development (GO:2000026)	3
30	Positive regulation of signal transduction (GO:0009967)	3
31	Regulation of signal transduction (GO:0009966)	3
32	Positive regulation of metabolic process (GO:0009893)	3
33	Positive regulation of biosynthetic process (GO:0009891)	3
34	Regulation of biosynthetic process (GO:0009889)	3
35	Response to stimulus (GO:0050896)	3
36	Positive regulation of response to stimulus (GO:0048584)	3
37	Regulation of response to stimulus (GO:0048583)	3
38	Negative regulation of cellular process (GO:0048523)	3
39	Positive regulation of cellular process (GO:0048522)	3
40	Negative regulation of biological process (GO:0048519)	3
41	Positive regulation of biological process (GO:0048518)	3
42	Positive regulation of RNA metabolic process (GO:0051254)	3
43	Regulation of RNA metabolic process (GO:0051252)	3
44	Regulation of multicellular organismal process (GO:0051239)	3
45	Regulation of developmental process (GO:0050793)	3
46	Positive regulation of nitrogen compound metabolic process (GO:0051173)	3
47	Regulation of nitrogen compound metabolic process (GO:0051171)	3
48	Response to biotic stimulus (GO:0009607)	3
49	Response to external stimulus (GO:0009605)	3
50	Positive regulation of developmental process (GO:0051094)	3
51	Negative regulation of developmental process (GO:0051093)	3
52	Defense response to other organism (GO:0098542)	3
53	Positive regulation of cellular biosynthetic process (GO:0031328)	3
54	Regulation of cellular biosynthetic process (GO:0031326)	3
55	Positive regulation of cellular metabolic process (GO:0031325)	3
56	Regulation of cellular metabolic process (GO:0031323)	3
57	Response to external biotic stimulus (GO:0043207)	3
58	Macromolecule metabolic process (GO:0043170)	3
59	Positive regulation of cell communication (GO:0010647)	3
60	Regulation of cell communication (GO:0010646)	3
61	Positive regulation of macromolecule metabolic process (GO:0010604)	3
62	Positive regulation of signaling (GO:0023056)	3
63	Signaling (GO:0023052)	3
64	Regulation of signaling (GO:0023051)	3
65	Positive regulation of macromolecule biosynthetic process (GO:0010557)	3
66	Regulation of macromolecule biosynthetic process (GO:0010556)	3
67	Regulation of gene expression (GO:0010468)	3
68	Metabolic process (GO:0008152)	3
69	Positive regulation of transcription by RNA polymerase II (GO:0045944)	3
70	Positive regulation of nucleobase-containing compound metabolic process (GO:0045935)	3
71	Positive regulation of DNA-templated transcription (GO:0045893)	3
72	Positive regulation of cell adhesion (GO:0045785)	2
73	Regulation of angiogenesis (GO:0045765)	2
74	Immune response (GO:0006955)	2
75	Inflammatory response (GO:0006954)	2
76	Positive regulation of kinase activity (GO:0033674)	2
77	Response to cytokine (GO:0034097)	2
78	Positive regulation of cell differentiation (GO:0045597)	2
79	Negative regulation of cell differentiation (GO:0045596)	2
80	Regulation of cell differentiation (GO:0045595)	2
81	Response to organic substance (GO:0010033)	2
82	Positive regulation of leukocyte cell-cell adhesion (GO:1903039)	2
83	Regulation of leukocyte cell-cell adhesion (GO:1903037)	2
84	Multicellular organism development (GO:0007275)	2
85	Cell-cell signaling (GO:0007267)	2
86	Cytokine-mediated signaling pathway (GO:0019221)	2
87	Regulation of phosphate metabolic process (GO:0019220)	2
88	Positive regulation of protein phosphorylation (GO:0001934)	2
89	Regulation of protein phosphorylation (GO:0001932)	2
90	Regulation of cell development (GO:0060284)	2
91	Immune system process (GO:0002376)	2
92	Regulation of molecular function (GO:0065009)	2
93	Regulation of biological quality (GO:0065008)	2
94	Positive regulation of cytokine production (GO:0001819)	2
95	Regulation of cytokine production (GO:0001817)	2
96	Positive regulation of mononuclear cell proliferation (GO:0032946)	2
97	Regulation of mononuclear cell proliferation (GO:0032944)	2
98	Response to molecule of bacterial origin (GO:0002237)	2
99	Cell activation (GO:0001775)	2
100	Positive regulation of T-helper 1 cell cytokine production (GO:2000556)	2
101	Regulation of T-helper 1 cell cytokine production (GO:2000554)	2
102	Positive regulation of type II interferon production (GO:0032729)	2
103	Positive regulation of granulocyte macrophage colony-stimulating factor production (GO:0032725)	2
104	Positive regulation of chemokine production (GO:0032722)	2
105	Response to organic cyclic compound (GO:0014070)	2
106	Positive regulation of phosphatidylinositol 3-kinase signaling (GO:0014068)	2
107	Regulation of phosphatidylinositol 3-kinase signaling (GO:0014066)	2
108	Cellular response to organic cyclic compound (GO:0071407)	2
109	Cellular response to oxygen-containing compound (GO:1901701)	2
110	Response to oxygen-containing compound (GO:1901700)	2
111	Regulation of type II interferon production (GO:0032649)	2
112	Regulation of granulocyte macrophage colony-stimulating factor production (GO:0032645)	2
113	Regulation of chemokine production (GO:0032642)	2
114	Cellular response to lipid (GO:0071396)	2
115	Cellular response to cytokine stimulus (GO:0071345)	2
116	Cellular response to organic substance (GO:0071310)	2
117	Cellular response to chemical stimulus (GO:0070887)	2
118	Regulation of organelle organization (GO:0033043)	2
119	Multicellular organismal process (GO:0032501)	2
120	Cellular response to lipopolysaccharide (GO:0071222)	2
121	Cellular response to molecule of bacterial origin (GO:0071219)	2
122	Cellular response to biotic stimulus (GO:0071216)	2
123	Response to lipopolysaccharide (GO:0032496)	2
124	Regulation of cell motility (GO:2000145)	2
125	Regulation of locomotion (GO:0040012)	2
126	Regulation of cellular response to growth factor stimulus (GO:0090287)	2
127	Positive regulation of leukocyte proliferation (GO:0070665)	2
128	Regulation of leukocyte proliferation (GO:0070663)	2
129	System development (GO:0048731)	2
130	Aminoglycan metabolic process (GO:0006022)	2
131	Negative regulation of signal transduction (GO:0009968)	2
132	Regulation of vasculature development (GO:1901342)	2
133	Primary metabolic process (GO:0044238)	2
134	Cellular metabolic process (GO:0044237)	2
135	Positive regulation of catabolic process (GO:0009896)	2
136	Regulation of catabolic process (GO:0009894)	2
137	Positive regulation of transferase activity (GO:0051347)	2
138	Regulation of transferase activity (GO:0051338)	2
139	Positive regulation of NIK/NF-kappab signaling (GO:1901224)	2
140	Regulation of NIK/NF-kappab signaling (GO:1901222)	2
141	Negative regulation of response to stimulus (GO:0048585)	2
142	Positive regulation of T cell activation (GO:0050870)	2
143	Positive regulation of cell activation (GO:0050867)	2
144	Regulation of cell activation (GO:0050865)	2
145	Regulation of T cell activation (GO:0050863)	2
146	Defense response to Gram-positive bacterium (GO:0050830)	2
147	Animal organ development (GO:0048513)	2
148	Positive regulation of response to external stimulus (GO:0032103)	2
149	Regulation of response to external stimulus (GO:0032101)	2
150	Programmed cell death (GO:0012501)	2
151	Positive regulation of lymphocyte activation (GO:0051251)	2
152	Regulation of lymphocyte activation (GO:0051249)	2
153	Positive regulation of protein metabolic process (GO:0051247)	2
154	Regulation of protein metabolic process (GO:0051246)	2
155	Negative regulation of multicellular organismal process (GO:0051241)	2
156	Positive regulation of multicellular organismal process (GO:0051240)	2
157	Lipopolysaccharide-mediated signaling pathway (GO:0031663)	2
158	Carbohydrate derivative metabolic process (GO:1901135)	2
159	Regulation of catalytic activity (GO:0050790)	2
160	Positive regulation of immune response (GO:0050778)	2
161	Regulation of immune response (GO:0050776)	2
162	Positive regulation of molecular function (GO:0044093)	2
163	Positive regulation of inflammatory response (GO:0050729)	2
164	Regulation of inflammatory response (GO:0050727)	2
165	Regulation of phosphorus metabolic process (GO:0051174)	2
166	Regulation of kinase activity (GO:0043549)	2
167	Positive regulation of cellular component organization (GO:0051130)	2
168	Regulation of cellular component organization (GO:0051128)	2
169	Anatomical structure morphogenesis (GO:0009653)	2
170	Response to bacterium (GO:0009617)	2
171	Positive regulation of lymphocyte proliferation (GO:0050671)	2
172	Regulation of lymphocyte proliferation (GO:0050670)	2
173	Positive regulation of NF-kappab transcription factor activity (GO:0051092)	2
174	Positive regulation of DNA-binding transcription factor activity (GO:0051091)	2
175	Regulation of DNA-binding transcription factor activity (GO:0051090)	2
176	Macromolecule modification (GO:0043412)	2
177	Positive regulation of protein modification process (GO:0031401)	2
178	Protein modification process (GO:0036211)	2
179	Regulation of protein modification process (GO:0031399)	2
180	Positive regulation of defense response (GO:0031349)	2
181	Regulation of defense response (GO:0031347)	2
182	Positive regulation of cellular catabolic process (GO:0031331)	2
183	Regulation of cellular catabolic process (GO:0031329)	2
184	MAPK cascade (GO:0000165)	2
185	Defense response to bacterium (GO:0042742)	2
186	Intracellular signal transduction (GO:0035556)	2
187	Positive regulation of catalytic activity (GO:0043085)	2
188	Homeostatic process (GO:0042592)	2
189	Positive regulation of neuroinflammatory response (GO:0150078)	2
190	Regulation of neuroinflammatory response (GO:0150077)	2
191	Positive regulation of phosphorylation (GO:0042327)	2
192	Regulation of phosphorylation (GO:0042325)	2
193	Regulation of cell migration (GO:0030334)	2
194	Positive regulation of cell development (GO:0010720)	2
195	Response to chemical (GO:0042221)	2
196	Negative regulation of cell communication (GO:0010648)	2
197	Regulation of anatomical structure morphogenesis (GO:0022603)	2
198	Positive regulation of organelle organization (GO:0010638)	2
199	Glycosaminoglycan metabolic process (GO:0030203)	2
200	Positive regulation of gene expression (GO:0010628)	2
201	Negative regulation of signaling (GO:0023057)	2
202	Positive regulation of cell population proliferation (GO:0008284)	2
203	Regulation of T cell proliferation (GO:0042129)	2
204	Regulation of cell population proliferation (GO:0042127)	2
205	Regulation of cell adhesion (GO:0030155)	2
206	Positive regulation of phosphorus metabolic process (GO:0010562)	2
207	Positive regulation of T cell proliferation (GO:0042102)	2
208	Cell death (GO:0008219)	2
209	Mucopolysaccharide metabolic process (GO:1903510)	2
210	Positive regulation of cell-cell adhesion (GO:0022409)	2
211	Regulation of cell-cell adhesion (GO:0022407)	2
212	Positive regulation of T-helper 1 type immune response (GO:0002827)	2
213	Regulation of T-helper 1 type immune response (GO:0002825)	2
214	Positive regulation of adaptive immune response based on somatic recombination of immune receptors built from immunoglobulin superfamily domains (GO:0002824)	2
215	Regulation of adaptive immune response based on somatic recombination of immune receptors built from immunoglobulin superfamily domains (GO:0002822)	2
216	Positive regulation of adaptive immune response (GO:0002821)	2
217	Response to lipid (GO:0033993)	2
218	Regulation of adaptive immune response (GO:0002819)	2
219	Positive regulation of phosphate metabolic process (GO:0045937)	2
220	Positive regulation of T cell cytokine production (GO:0002726)	2
221	Regulation of T cell cytokine production (GO:0002724)	2
222	Positive regulation of cytokine production involved in immune response (GO:0002720)	2
223	Positive regulation of protein kinase activity (GO:0045860)	2
224	Regulation of cytokine production involved in immune response (GO:0002718)	2
225	Regulation of protein kinase activity (GO:0045859)	2
226	Positive regulation of T cell mediated immunity (GO:0002711)	2
227	Regulation of T cell mediated immunity (GO:0002709)	2
228	Positive regulation of lymphocyte mediated immunity (GO:0002708)	2
229	Regulation of lymphocyte mediated immunity (GO:0002706)	2
230	Positive regulation of leukocyte mediated immunity (GO:0002705)	2
231	Regulation of leukocyte mediated immunity (GO:0002703)	2
232	Positive regulation of production of molecular mediator of immune response (GO:0002702)	2
233	Regulation of production of molecular mediator of immune response (GO:0002700)	2
234	Protein metabolic process (GO:0019538)	2
235	Regulation of response to stress (GO:0080134)	2
236	Positive regulation of immune effector process (GO:0002699)	2
237	Regulation of immune effector process (GO:0002697)	2
238	Positive regulation of leukocyte activation (GO:0002696)	2
239	Regulation of leukocyte activation (GO:0002694)	2
240	Positive regulation of immune system process (GO:0002684)	2
241	Regulation of immune system process (GO:0002682)	2

Uploading the core genes to the KEGG database, we obtained 17 pathways that involve at least two core genes (Table 4). Out of these, 10 pathways were related to infectious diseases, and 7 pathways were related to immunity and inflammation. These results demonstrate that immunity and inflammation play significant roles in the common pathogenesis and cross-talk pathways of PD and AD.

**TABLE 4:** KEGG analysis of core genes.

	KEGG Mapper Search Result	SUM	DCN	IL18	IL1B	MLKL
1	hsa04623 Cytosolic DNA-sensing pathway	3	0	1	1	1
2	hsa05132 Salmonella infection	3	0	1	1	1
3	hsa04217 Necroptosis	2	0	0	1	1
4	hsa05134 Legionellosis	2	0	1	1	0
5	hsa05146 Influenza A	2	0	1	1	0
6	hsa05143 African trypanosomiasis	2	0	1	1	0
7	hsa05144 Malaria	2	0	1	1	0
8	hsa05323 Rheumatoid arthritis	2	0	1	1	0
9	hsa05417 Lipid and atherosclerosis	2	0	1	1	0
10	hsa05152 Tuberculosis	2	0	1	1	0
11	hsa04621 NOD-like receptor signaling pathway	2	0	1	1	0
12	hsa04060 Cytokine-cytokine receptor interaction	2	0	1	1	0
13	hsa05135 Yersinia infection	2	0	1	1	0
14	hsa05130 Pathogenic Escherichia coli infection	2	0	1	1	0
15	hsa04668 TNF signaling pathway	2	0	0	1	1
16	hsa05131 Shigellosis	2	0	1	1	0
17	hsa05321 Inflammatory bowel disease	2	0	1	1	0

### 3.9 Immune infiltration

**FIGURE 12:**
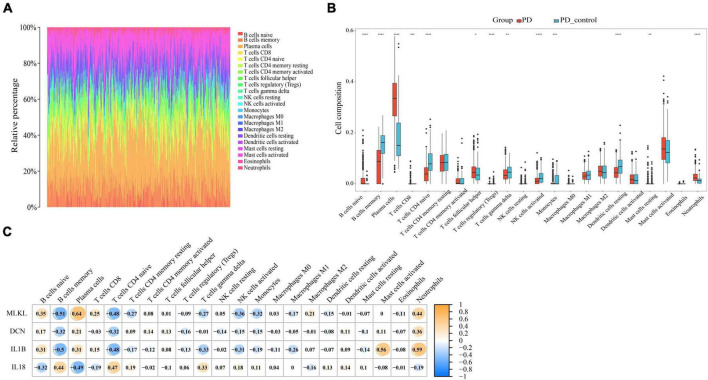
**(A)** Immune infiltration of each sample in PD; **(B)** Boxplots of each immune cell’s expression in PD (*P-value < 0.05, **P-value < 0.01, ***P-value < 0.001, ****P-value < 0.0001); **(C)** correlation between immune cells and core genes in PD.

**FIGURE 13:**
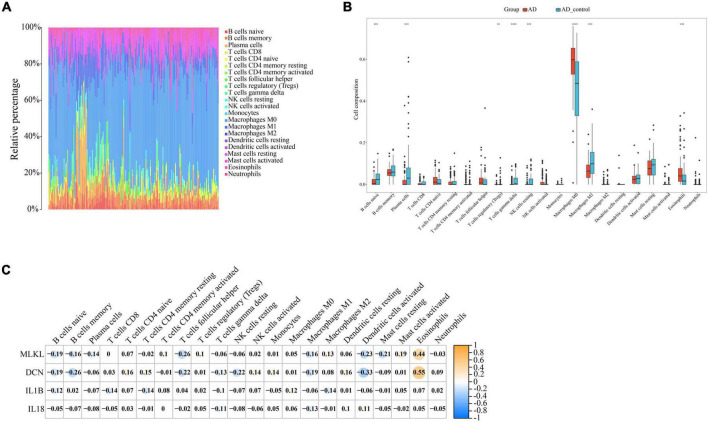
**(A)** Immune infiltration of each sample in AD; **(B)** Boxplots of each immune cell’s expression in AD (**P-value < 0.01, ***P-value < 0.001, ****P-value < 0.0001); **(C)** correlation between immune cells and core genes in AD.

Through our preceding analyses, we have identified a close association between PD and AD with the immune system. Consequently, we employed the CIBERSORT algorithm to analyze immune infiltration in the PD and AD datasets, aiming to further explore the role of immune cells in PD and AD, and the impact of key genes on them. In 22 immune cell subpopulations, we discovered substantial changes between PD and AD with normal tissues (P-value < 0.05). Figures 12B, 13B show that B cells naive, Plasma cells, and T cells gamma delta were changed to a greater extent in both PD and AD patients. As shown in Figures 12C, 13C, 4 core genes were associated with each immune cell. Correlation analysis was used to examine the correlation between immune cells and the core genes.

### 3.10 Drug-gene interaction analysis

Using the core genes, we identified 10 potential therapeutic drugs for Alzheimer’s disease patients with periodontitis (Table 5). Two drugs are associated with DCN, two drugs are associated with IL1B, two drugs are associated with MLKL, and four drugs are associated with IL18.

**TABLE 5:** Prediction of potential therapeutic agents.

	Gene	Drug	Interaction score
1	MLKL	COMPOUND 56	26.25383562
2	MLKL	MLKL COMPOUND 1	8.751278539
3	IL18	GSK-1070806	8.290684932
4	IL18	CAMOTESKIMAB	5.527123288
5	IL18	SYNTHETIC HUMAN PAPILLOMAVIRUS 16 E6 PEPTIDE	5.527123288
6	DCN	CM-352	3.750547945
7	IL1B	GEVOKIZUMAB	3.088686543
8	IL18	TADEKINIG ALFA	2.763561644
9	IL1B	CANAKINUMAB	2.573905453
10	DCN	MMP13 TRACER [18F]5J	2.500365297

## 4 Discussion

In this study, we downloaded the PD datasets (GSE10334 and GSE16134) and AD datasets (GSE5281 and GSE28146) from the GEO database. Next, we analyzed the differences between the PD and AD datasets separately. We used “Alzheimer’s disease” as the keyword in the DisGeNet database for text mining to obtain AD-related genes. The AD-related genes were merged into AD DEGs. Then, by crossing these two DEG sets, we obtained common DEGs for PD and AD, which were subsequently analyzed by GO and KEGG enrichment. Our results showed that the common DEGs were mainly enriched in inflammatory, immune, and other related pathways, suggesting a possible relationship between these two inflammation-related diseases. By constructing a PPI network, we identified 10 hub genes. Our speculations were validated by a more in-depth enrichment analysis of these hub genes. Next, 10 genes associated with PANoptosis were screened from the common DEGs of PD and AD. To further validate whether these genes could serve as potential PD diagnostic or therapeutic markers, we evaluated the ROC diagnostic value of cross-talk-PANoptosis genes. Four core genes (DCN, MLKL, IL1B, IL18) with high diagnostic value were finally identified.

Recent studies have found that the presence of different patterns of cell death has multiple critical implications for the immune system and its impact on health and disease (Fuchs and Steller, 2015; Bertheloot et al., 2021). Programmed cell death is a complex mechanism. Patterns of cell death are not completely independent and there is extensive crosstalk between them (Kesavardhana et al., 2020; Malireddi et al., 2020). PANoptosis is a uniquely integrated mode of cell death that is characterized by cellular pyroptosis, apoptosis, and necroptosis. Activation of PANoptosis is a common host immune response against infection (Pandian and Kanneganti, 2022). Therefore, the potential role of PANoptosis and its related genes in PD and AD is worth exploring.

A total of four genes were identified in our study to elucidate the link between PD and AD. Decorin (DCN), a leucine-rich small-molecule proteoglycan in the extracellular matrix (ECM), regulates a variety of cellular processes including collagen fibrillogenesis, wound repair, vascular arrest, tumor growth, and autophagy (Gubbiotti et al., 2016). Also known as PG40, it is not only a structural component of the ECM but also a key player in cellular signaling. It can be involved in various cell signaling pathways closely related to fibrosis and cancer (Wang H. et al., 2024; Lin et al., 2023), and also plays an important role in immunity and inflammation (Bocian et al., 2013; Frey et al., 2013; Borges et al., 2015). The current study found that decorin binds to TLR2 and TLR4 on macrophages with an affinity comparable to that of pathogen-derived ligands. This resulted in rapid activation of the p38, MAPK, and NF-κB pathways and enhanced synthesis of the pro-inflammatory cytokines TNF-α and IL-12 (Merline et al., 2011). DCN activates autophagy through AKT/mTOR/p70S6K signaling while inhibiting inflammation-induced apoptosis and ECM degradation (Gubbiotti et al., 2018; Zhang et al., 2020), demonstrating the close relationship between DCN and programmed cell death and PANoptosis, and confirming its potential role in PD and AD.

Necroptosis is a programmed form of cell death associated with a wide range of human pathological conditions. Mixed lineage kinase domain-like protein (MLKL) is a central effector of Necroptosis and its role in Necroptosis has been extensively studied (Cao et al., 2018; Garnish et al., 2021; Chen et al., 2013). In vivo and in vitro studies have shown that MLKL expression is significantly upregulated in gingival tissues of PD patients, and its mediated Necroptosis exacerbates the progression of PD (Yang et al., 2021), which is consistent with our analysis. Inhibition of RIPK3/MLKL-mediated Necroptosis in gingival fibroblasts by application of MLKL inhibitors (e.g., sh-MLKL) attenuates PD (Zhang K. et al., 2023). On the other hand, in AD-related studies, hyperphosphorylated tau was found to induce Necroptosis in neuronal cells by promoting the formation of RIPK1/RIPK3/MLKL necrosomes (Dong et al., 2022). In addition, a significant increase in the ratio of Aβ42/Aβ40 was observed in MLKL knockdown cells (Wang et al., 2018), and an elevated ratio of Aβ42/Aβ40 leads to an increased susceptibility to AD in the population (Pauwels et al., 2012). MLKL may become a key target for effective preventive therapy in PD and AD.

Proinflammatory cytokines, including IL1, IL2, IL12, IL17, IL18, IFNγ, and TNFα. which tightly regulate cell-mediated immune responses, and play an important role in modulating the immune system (Turner et al., 2014). In our study, the inflammatory cytokines IL1B and IL18 play important roles in the crosstalk pathway between PD and AD. Many studies have reported that IL1B polymorphisms affect susceptibility to periodontal disease and its progression (Walther et al., 2022). As a proinflammatory cytokine, IL1B is involved in inflammation, immunomodulation, and bone resorption in PD. Elevated levels of IL1B are frequently detected in saliva and gingival crevicular fluid (GCF) of PD patients compared to healthy controls (Demirci et al., 2023; Kainat et al., 2023). In addition, patients with chronic PD also reach higher levels of serum IL1B, inducing systemic effects (Engebretson et al., 2002). IL1B at the site of inflammation leads to increased local blood flow, leukocyte recruitment, and neutrophil infiltration. Matrix metalloproteinase (MMP)-9 is an important indicator of the severity and progression of PD (Luchian et al., 2022). IL1B increases the expression of collagenolytic enzymes, MMP, which contributes to extracellular matrix degradation, which in turn leads to bone resorption and tissue destruction (Kobayashi et al., 2005; Galozzi et al., 2021). IL1B is also involved in the up-regulation of receptor activator for nuclear factor-κ ligand (RANKL), which stimulates osteoclastogenesis (Huynh et al., 2017). In peripheral inflammation, cytokines such as TNFα, IL6, and IL1B can affect and cross the blood-brain barrier (BBB), stimulating its release of proinflammatory mediators and making it more permeable to cells, thereby allowing leukocytes to pass through the brain (Galea, 2021). These phenomena lead to microglial and astrocytic responses in the brain involving further production of pro-inflammatory mediators, reactive oxygen species (ROS), and reactive nitrogen species (RNS), among others. This mechanism has been defined as neuroinflammation (Novoa et al., 2022). Theoretically, chronic neuroinflammation may be the initial molecular pathology leading to neuronal death in AD (Yushen Yushen Li et al., 2022). Previous studies have shown that IL18 is involved in the activation of not only Th1 cells, and NK cells, but also Th2, IL17-producing γδ T cells and macrophages (Kaplanski, 2017). Thus, IL18 is an important regulator of different types of immune cells. The current findings suggest that the proinflammatory function of IL18 has an impact on the inflammatory response within periodontal tissues (Zhang et al., 2021). In vitro studies have shown that IL18 stimulation does not affect the cell cycle, apoptosis, and proliferation of healthy periodontal cells, but significantly promotes the production of proinflammatory cytokines by periodontal cells, and that IL18 may contribute to periodontal disease, either directly or indirectly, by promoting the migration of inflammatory cells (Yucel-Lindberg and Båge, 2013). Infiltration of inflammatory cells requires specific cellular and protein mediators, including neutrophils, chemokines, and MMP (Sochalska and Potempa, 2017; Nagashima et al., 2017; Mauramo et al., 2017). Abnormal upregulation of IL18 has been observed in neurons and glial cells of AD patients (Yamanishi et al., 2023). IL18 promotes Aβ production and kinase activity, which is important for tau phosphorylation (Sutinen et al., 2012).

To gain a deeper understanding of the underlying mechanisms linking PD and AD, this study identified signaling pathways enriched by core genes. The Cytosolic DNA-sensing pathway plays a crucial role in the regulation of PD and AD. The Cytosolic DNA-sensing cGAS-STING pathway plays a crucial role in immune defense against various DNA viruses, retroviruses, and bacteria (Chen et al., 2016). It has been demonstrated that Porphyromonas gingivalis infection promotes the progression of the inflammatory response through the cGAS-STING signaling pathway in human gingival fibroblasts (HGFs) and a mouse model of PD (Bi et al., 2023). In addition, insulin-like growth factor 2 (IGF2) can inhibit M1 macrophage polarization through the cGAS-STING pathway and promote osteogenesis in a mouse model of PD (Wang T. et al., 2024). Biomarkers of AD-associated neuritis include NF-κB and cGAS-STING pathway (Guo et al., 2024). It was found that cGAS-STING was elevated in the brains of AD mice and human AD fibroblasts (Chen K. et al., 2022). Nicotinamide riboside (NR) reduces neuroinflammation and cellular senescence and improves cognitive function in AD mice by modulating the cGAS-STING pathway (Hou et al., 2021).

The results of the pathway analysis suggest that the immune system is the main mechanism linking PD and AD. Changes in immune cells seem to play a greater role in the relationship between PD and AD. It has been shown that NK cells and MDSCs are highly expressed in both PD and AD (Yang et al., 2024). In PD, NK cells are involved in the immune response to oral pathogens. In addition, NK cells produce cytokines, including proinflammatory cytokines (Kikuchi et al., 2005). NK cells have been detected in the brains of AD patients, particularly in areas affected by neurodegeneration and Aβ plaque deposition (Lu et al., 2021). In addition, it has been shown that supplementation with exogenous MDSCs can significantly treat neuroinflammation and cognitive deficits (Cheng et al., 2023).

Based on the immune infiltration landscape results between PD and AD, we found significant differences in the expression of B cells naïve, plasma cell and T cells gamma delta in patients with PD and AD. Previous studies have shown that B cells naïve are involved in periodontal immunomodulation (Li et al., 2020). However, current data suggest that the role and function of B cells naïve in periodontitis is unclear. Therefore, further studies are needed to understand the involvement of periodontal immunomodulation in periodontitis. Plasma cells have the ability and capacity to produce and release antibodies to kill microorganisms in the tissues (Mizutani et al., 2016). When bacteria invade the connective tissues of the gingiva, B cells are activated and become plasma cells to produce antibodies (Mizutani et al., 2014). T cells gamma delta are a special type of lymphocyte that can participate in both innate and adaptive immune responses (Lee et al., 2020). T cells gamma delta are likely involved in the physiological and pathological processes of bone in periodontitis, yet their role in the pathology of human oral diseases remains elusive (Chen et al., 2023). Due to the blood-brain barrier, the brain is considered immunologically privileged and few peripheral immune cells are detected in the brain parenchyma (Jevtic et al., 2017). Macrophages (microglia) are resident innate immune cells of the central nervous system and play a key role in maintaining immune defense and homeostasis in the body (Madore et al., 2020). In the present study, we found significantly lower B cells naive, Plasma cells, and T cells gamma delta scores in the AD group and higher scores for Eosinophils and macrophages in AD. This result supports the important role of innate immunity in the development of AD. Innate immune cell hyperexcitability was reported to be associated with cognitive decline (Nam et al., 2022).

In summary, based on the current findings, we can hypothesize three potential relationships between PD and AD: (I) Local inflammation in patients with periodontitis can cause changes in the levels of B-cells, T-cells and neutrophils in the circulatory system, which in turn affects the fragile immune balance in the brain. (II) Cognitive decline and differential expression of core genes in AD patients can lead to changes in the content of immune cells in gingival tissues, increasing the risk of PD or exacerbating pre-existing PD symptoms. (III) Core genes may affect PD and AD by influencing PANoptosis and innate immunity, and participating in the cGAS-STING pathway.

By screening the DGIDB database, four core genes were applied to predict drug candidates, and 10 drugs of interest were screened. Compound 56 partially inhibits MLKL oligomerization and significantly inhibits its translocation to the membrane (Cui et al., 2022). MLKL Compound 1 specifically binds to both cysteines of heat shock protein 70 (HSP70), thereby blocking its function. Importantly, HSP70 promotes MLKL polymerization to activate necroptosis (Johnston and Wang, 2020). Camoteskimab is a high-affinity, fully human anti-IL-18 monoclonal antibody being developed for the treatment of autoinflammatory diseases (Galozzi et al., 2022). Canakinumab, a human monoclonal anti-IL-1β antibody that neutralizes human IL-1β activity and inhibits inflammation, is now in clinical use and is currently approved for the treatment of different auto-inflammatory diseases (Chakraborty et al., 2012). A randomized, placebo-controlled phase II clinical trial on canakinumab has recently been registered in patients with mild cognitive impairment or mild AD with evidence of peripheral inflammation. These tests provide a comprehensive assessment of memory, attention and verbal fluency as affected by early AD. In addition, the effects of the drug on central and peripheral inflammation will be assessed (Arbo et al., 2022).

This study considers, for the first time, the role of cellular PANoptosis-related genes in PD and AD, and uses bioinformatics to explore the link between them. As a risk factor for AD, PD can produce a range of systemic inflammatory responses in the host. Preventing PD could prevent the development of AD in the population while reducing the incidence of PD. The biomarkers we identified can enable effective diagnosis of PD disease and play an active role in early predictive prevention of AD. The application of new tools such as machine learning and community discovery made this study more comprehensive and novel.

## 5 Conclusion

In this study, four core genes, DCN, MLKL, IL1B, and IL18, were utilized to reveal a common mechanism associated with PANoptosis, supporting a strong interrelationship between PD and AD. Targeted drugs were screened based on the four core genes, providing a new strategy for potential drug treatment of PD and AD. In addition, immune infiltration in patient tissues explored the relationship between PD and AD and the relationship between key markers and immune cells. Our findings may guide future studies on the molecular mechanisms underlying the relationship between PD and AD and may provide new potential targets for diagnosis and treatment.

## Data Availability

Publicly available datasets were analyzed in this study. This data can be found here: https://www.ncbi.nlm.nih.gov/geo/.

## Footnotes

1
https://www.ncbi.nlm.nih.gov/geo/

2
https://www.disgenet.org/

3
http://www.string-db.org/

4
http://www.dgidb.org
